# [Retracted] Effect of selective inhibition or activation of PGE2 EP1 receptor on glomerulosclerosis

**DOI:** 10.3892/mmr.2025.13538

**Published:** 2025-04-15

**Authors:** Xu Chen, Jun Yin, Yuyin Xu, Zhi Qiu, Jing Liu, Xiaolan Chen

Mol Med Rep 22: 2887–2895, 2020; DOI: 10.3892/mmr.2020.11353

Following the publication of the above paper, it was drawn to the Editor's attention by a concerned reader that, regarding the immunohistochemical images shown in Fig. 2C and 3A, at least four pairs of data panels showed evidence of overlapping data, both within the same figure parts and comparing between them.

Owing to the large number of data duplication events that have been identified in this paper, the Editor of *Molecular Medicine Reports* has decided that it should be retracted from the Journal on account of a lack of confidence in the presented data. The authors were asked for an explanation to account for these concerns, but the Editorial Office did not receive a satisfactory reply. The Editor apologizes to the readership for any inconvenience caused.

